# Affective Disorders among Patients with Borderline Personality Disorder

**DOI:** 10.1371/journal.pone.0050930

**Published:** 2012-12-06

**Authors:** Hege Nordem Sjåstad, Rolf W. Gråwe, Jens Egeland

**Affiliations:** 1 Division of Mental Health and Addiction, Vestfold Hospital Trust, Tønsberg, Norway; 2 Department of Research and Development, Alcohol and Drug Treatment Health Trust in Central Norway, Trondheim, Norway; University of Iowa Hospitals & Clinics, United States of America

## Abstract

**Background:**

The high co-occurrence between borderline personality disorder and affective disorders has led many to believe that borderline personality disorder should be considered as part of an affective spectrum. The aim of the present study was to examine whether the prevalence of affective disorders are higher for patients with borderline personality disorder than for patients with other personality disorders.

**Methods:**

In a national cross-sectional study of patients receiving mental health treatment in Norway (N = 36 773), we determined whether psychiatric outpatients with borderline personality disorder (N = 1 043) had a higher prevalence of affective disorder in general, and whether they had an increased prevalence of depression, bipolar disorder or dysthymia specifically. They were compared to patients with paranoid, schizoid, dissocial, histrionic, obsessive-compulsive, avoidant, dependent, or unspecified personality disorder, as well as an aggregated group of patients with personality disorders other than the borderline type (N = 2 636). Odds ratios were computed for the borderline personality disorder group comparing it to the mixed sample of other personality disorders. Diagnostic assessments were conducted in routine clinical practice.

**Results:**

More subjects with borderline personality disorder suffered from unipolar than bipolar disorders. Nevertheless, borderline personality disorder had a lower rate of depression and dysthymia than several other personality disorder groups, whereas the rate of bipolar disorder tended to be higher. Odds ratios showed 34% lower risk for unipolar depression, 70% lower risk for dysthymia and 66% higher risk for bipolar disorder in patients with borderline personality disorder compared to the aggregated group of other personality disorders.

**Conclusions:**

The results suggest that borderline personality disorder has a stronger association with affective disorders in the bipolar spectrum than disorders in the unipolar spectrum. This association may reflect an etiological relationship or diagnostic overlapping criteria.

## Introduction

Traditionally, borderline personality disorder (BPD) has been considered as a threshold psychotic disorder [1]. Recently, it has instead been associated with affective disorders [2]. Affective disorders are common among patients with BPD [3]-[5] and high rates of BPD have repeatedly been found among patients with depression [6]-[8]. However, a high rate of comorbidity is not sufficient to conclude that affective disorders are a part of the BPD condition. High rates of affective disorders among persons with BPD may reflect several conditions; 1) there is generally a high occurrence of affective disorders in patient populations, 2) patients with personality disorders, regardless of type, may be more likely to have an affective disorder than patients with other disorders, and/or 3) there may be an unique clinical relationship between BPD and affective disorders.

There is a huge variation in prevalence figures between studies of BPD among patients with major depression. Research findings vary between 7% and 63% [6]-[8]. This variation could be explained by sample variation, (eg. inpatient versus outpatient status) and the use of different assessment methods. Brieger, Ehrt and Marneros [9] reported that between 7% and 30% of patients with bipolar affective disorders also had BPD. The rate of BPD among patients with dysthymia has been found to vary between 5% [10] and 24% [11].

McGlashan et al. [4] found that 71% of patients with BPD had a current lifetime prevalence of depression, and Zanarini et al. [5] reported a comparable point prevalence of 87%. The same studies observed that between 17% and 45% of the patients with BPD fulfilled the criteria for dysthymia, and that between 6% and 20% fulfilled the criteria for bipolar affective disorder. Deltito et al. [3] found that 45% of patients with BPD had a co-occurring bipolar affective disorder.

Even though there seems to be a high comorbidity between affective disorders and BPD, it is no current consensus as to how this should be understood. Some researchers suggest that BPD should be viewed as an affective spectrum disorder, and are consequently reluctant to classify BPD as a personality disorder [12],[13]. They refer to the high co-occurrence between BPD and bipolar spectrum disorders, positive family histories for both disorders, and shared phenomenology and biological markers. Other researchers claim that the co-occurrence between BPD and affective disorders is no more specific than to other personality disorders [14],[15]. They refer to studies which have found that the defining clinical features of BPD are interpersonal [16], that individuals with BPD have mood swings qualitatively different from those with bipolar disorder [17], that BPD requires specialized treatment programs [18],[19] and do not respond well to mood stabilizers [16],[19]. It is also documented that changes in BPD and affective symptoms interact [20],[18]. All these factors contribute to a conceptualization of BPD as an independent disorder with multiple axis I diagnoses.

How we understand the interaction between BPD and affective disorders has implications for how affective symptoms among patients with BPD are contextualized as either state or trait dependent, biologically or situationally determined. This may in turn influence the treatment offered to patients with BPD symptoms. On this basis, comparing the prevalence of affective disorders in BPD with other personality disorders may contribute further to our understanding of the association between affective spectrum disorders and BPD. To our knowledge, only a few studies have examined this issue. Zanarini et al. [5] found significantly higher prevalence of general affective disorders in patients with BPD than in a group of persons suffering from other personality disorders. However, Fyer, Frances, Sullivan, Hurt, and Clarkin [21] and Zimmerman and Coryell [22] (comparing relatives of healthy controls and psychiatric patients), found no such difference. McGlashan et al. [4] and Perry [23] found no differences in the prevalence of different affective disorders among patients with BPD compared to a selection of other personality disorders.

Further studies comparing affective disorders in BPD and specific other personality disorders are needed, because comparisons between BPD and a mixed selection of other personality disorders may be misleading. Clinical samples with different personality disorders may represent an average with high or low prevalence of affective disorders. It is also essential to differentiate affective disorders into unipolar depression, bipolar affective disorder and dysthymia, because factors that distinguish the different personality disorders from one another (e.g. biological, psychosocial and temperamental) may interact with the various affective disorders in different ways. It is possible that persons with BPD are vulnerable to unipolar depression. An increased number of stressors related to impaired psychosocial functioning may, on the one hand, increase the risk of depression. This may, however, also be relevant for other personality disorders. On the other hand, there may be a more direct relationship between bipolar disorder and BPD than between bipolar disorder and other personality disorders. Thus, the varying results from studies exploring the relationship between affective disorders and BPD may be due to differing mechanisms mediating the different affective disorders.

### Aims of the Study

In order to provide a better understanding of the relationship between BPD and affective disorders, we wanted to compare the occurrence of affective disorders among patients with BPD and other types of personality disorders. The present study examines whether the prevalence of affective disorders in general, or unipolar depression, bipolar disorder, and dysthymia separately, are higher in persons with borderline personality disorder than in persons with other personality disorders.

## Materials and Methods

### Ethics Statement

Data was obtained from a cross-sectional study commissioned by the Norwegian Directorate of Health as part of the National Patient Registration in Norway in 2008. To ensure an accurate picture of the patient population, consent was not obtained during the registration process. No information that could identify the participants was recorded, therefor it has not been possible to obtain consent for the present study either. The study was approved by the Norwegian Regional Ethics Committee and the Norwegian Data Inspectorate.

### Data

Data was obtained from a cross-sectional study carried out by SINTEF Technology and Society, and commissioned by the Norwegian Directorate of Health as a part of the National Patient Registration in 2008. The data was collected from all Norwegian Community Mental Health Centres and all private practitioners (psychiatrists and clinical psychologists) with a licensed work agreement with the Norwegian Health Trusts. Demographic, clinical and treatment data were registered for all unique patients over age 18 receiving outpatient treatment during a 2-week period in 2008 (n = 36 773) [24]. Each patient was recorded only once regardless of their number of sessions in the inclusion period. Sixty-seven percent received treatment at the Community Mental Health Centres and the remainder from private practitioners. It is estimated that the study included 93% of all outpatients in Norway who were in contact with a Community Mental Health Centres during the registration period, and 66% of patients in private treatment [24].

### Assessment

The therapists assessed demographic and clinical information for each patient, and included one primary and one secondary diagnosis listed in the 10^th^ revision of the International Classification of Diseases (ICD-10) [25]. Diagnosis was based on routine clinical assessments. It is not known how many clinicians used structured diagnostic interviews.

### Sample

All patients with personality disorder diagnosis (n = 3 752) were identified in the main study data set. In order to allow comparisons of affective and personality disorders, subjects with more than one personality disorder (n = 73) were excluded. Thus, the sample consisted of 3 679 outpatients with one registered personality disorder, wherein 70% had a personality disorder as their primary diagnosis. A total of 946 subjects had an unspecified personality disorder. A non BPD personality disorder group (n = 2 636) was computed in order to perform comparisons with other studies dichotomizing their samples into BPD versus other personality disorders [5],[21],[22]. In the total sample (n = 3 679) 68% were female, the average age was 39 years (SD = 11.6, range 18), and 64% were living alone. Educational level was rated on a five point scale (1 = incomplete primary school, 2 = completed primary school, 3 = completed high school, 4 = university BA, 5 = university MA). Most frequent educational level (mode) for this sample was high school. Median length of current treatment period was 15 months. Among subjects included in this study (n = 3 679), 4% had a substance abuse disorder and 1.4% had a psychotic disorder.

### Dependent Variables

The following affective disorders were classified: *bipolar disorder* (F31.0– F31.9 in ICD-10 [25]), *depression* (F32.0– F33.9), *dysthymia* (F34.1) and *affective disorder* including all affective disorders in ICD-10 (F30– F39). Among the participants, only seven were diagnosed with cyclothymia, therefore cyclothymia was not classified in a separate category.

Diagnosis in the schizophrenia spectrum, schizotypal and delusional disorders were categorized as psychotic disorders (F20– F29 in ICD-10). All disorders due to psychoactive substance use were categorized as substance use disorders (F10– F19 in ICD-10).

### Statistics

Student t-tests and Chi-Square tests were performed to determine whether there were differences between the BPD group and each of the personality disorder groups on demographic, clinical and various affective disorder variables. Because BPD is compared to nine different personality disorders (multiple comparisons), we adopted a.0025 alpha level. Odds ratios were computed for each affective disorder comparing the BPD group to the non-BPD personality disorder group. The confidence interval was set to 95%.

## Results

Demographic and clinical characteristics of the different personality disorders are presented in Table 1. Statistical tests showed that patients with BPD were significantly younger than patients with most other personality disorders, except for the schizoid and dissocial types. Eighty-six percent of the patients with BPD were female. Overall, patients with BPD were more likely to be female than patients with other personality disorders. This applied to all personality disorder groups except for the histrionic and dependent types. Patients with BPD had significantly lower education than the other personality disorder groups, apart from the dissocial and paranoid types. Seventy percent of the BPD patients were living alone. They were significantly more often living alone compared to all other personality disorder groups, except from paranoid, schizoid, dissocial and histrionic personality disorders. Mean length for current treatment episode for patients with BPD was 25 months. Apart from patients with dependent, histrionic and dissocial personality disorders (whose current treatment time was longer), there were only minor differences between the different PD groups. Relatively few patients with BPD had coexisting substance use disorders (5.8%) or psychotic disorders (1.8%). Apart from patients with avoidant personality disorder, the non-BPD personality disorder group (who both had significantly lower rates of substance use and psychotic disorders) and dissocial personality disorder (significantly higher rates of substance use and psychotic disorders), the prevalence of coexisting substance use or psychotic disorders in the BPD group did not differ significantly from patients with other personality disorders.

**Table 1:** Demographic and clinical composition of specific personality disorder groups and non-BPD personality disorder group.

	Personality disorder study groups										
	Borderline	Paranoid	Schizoid	Dissocial	Histrionic	OCPD^a^	Avoidant	Dependent	Unspecified	Non-BPD PD^b^	
	(n = 1043)	(n = 194)	(n = 106)	(n = 64)	(n = 45)	(n = 131)	(n = 918)	(n = 232)	(n = 946)	(n = 2636)	
Gender,female (%)	86	55*	51*	19*	80	51*	62*	79	62*	61*	
Age in years(Mean ± SD)	35.5±10.8	41.0*±12.7	38.5±12.3	35.5±10.4	47.2*±11.4	43.0*±10.7	40.3*±11.0	42.0*±11.3	40.0*±12.0	40.4*±11.6	
Living alone/notliving alone^c^ (%)	70/30	70/30	71/29	71/29	70/30	47/53*	59/41*	57/43*	62/38*	61/39*	
Education^d^(Mean ± SD)	2.8±0.8	2.9±0.9	3.1*±1.0	2.3*±0.8	3.3*±1.0	3.5*±0.9	3.0*±0.9	3.2*±0.9	3.2*±0.9	3.1*±0.9	
Treatment length(mo)(Mean ± SD)	25±34	27±33	35±43	18±24	32±30	36±47	28±34	36*±42	28±36	29*±36	
Substance usedisorder (%)	5.8	5.2	1.9	28.1*	4.4	.8	2.0*	2.6	3.1	3.3*	
Psychotic disorder (%)	1.8	2.1	.9	7.8*	4.4	.0	.4	.0	1.9	1.3	

a Obsessive-compulsive personality disorder.

b Non-BPD personality disorder.

c Living alone = not married, separated/divorced, widowed/widower; Not living alone = married, cohabitant.

d Education was rated on a five point scale: 1 = incomplete primary school, 2 = primary school, 3 = high school, 4 = university BA, 5 = university MA.

* Differ significantly from BPD group (p<.0025).

The rates of affective disorders among patients with BPD and other personality disorders are shown in Table 2. The BPD group had significantly lower prevalence of affective disorders than the avoidant type and the non-BPD group. With regard to depression, patients with BPD had significantly lower prevalence than the avoidant, dependant and unspecified types and the non-PBD personality disorder group. The BPD group had numerically lower occurrence of affective disorders and depression than all other groups, except for the dissocial type. With regard to bipolar disorder, patients with BPD had significantly higher prevalence than the non-BPD personality disorder group but did not differ significantly from the other personality disorders separately. Nevertheless, the BPD group had a numerically higher occurrence of bipolar disorder than all other groups, except for the dissocial and obsessive-compulsive personality disorder (OCPD) groups. Patients with BPD had significantly lower prevalence of dysthymia than patient with schizoid and avoidant personality disorders.

**Table 2:** Affective disorders among patients with BPD relative to patients with other personality disorders.

	Borderline	Paranoid	Schizoid	Dissocial	Histrionic	OCPD^a^	Avoidant	Dependent	Unspecified	Non-BPD PD^b^	
	(n = 1043)	(n = 194)	(n = 106)	(n = 64)	(n = 45)	(n = 131)	(n = 918)	(n = 232)	(n = 946)	(n = 2636)	
Affectivedisorder	%	%	%	%	%	%	%	%	%	%	OR†
Affectivedisorder^c^	22.0	26.8	32.1	12.5	22.2	32.8	32.4*	30.6	27.6	29.4*	.75
Depression	14.9	22.2	22.6	6.2	17.8	21.4	24.9*	26.7*	20.8*	22.6*	.66
Bipolardisorder	5.7	2.1	2.8	6.2	2.2	6.1	3.1	2.2	3.9	3.4*	1.66
Dystymicdisorder	.9	2.6	6.6*	.0	.0	3.1	4.2*	.9	2.3	3.0*	.29

a Obsessive-compulsive personality disorder.

b Non-BPD personality disorder.

c The percentages in the variable *affective disorder* are larger than the total of the subcategories depression, bipolar disorder, and dysthymia, because the variable includes all types of affective disorders listed in ICD-10 (F30–F39) [25].

* Differ significantly from BPD group (p<.0025).

† Odds ratio for the affective illnesses in the BPD group compared to non-BPD PD.

Odds ratios showed that patients with BPD had a 34% lower risk (OR = 0.66) for depression compared to the total sample of patients with non-BPD personality disorders. The risk for dysthymia among BPD subjects was one in three compared to the other personality disorders (OR = 0.29).With regard to the probability ratio for bipolar disorder, the direction of results was reversed, i.e. the BPD group had a 66% higher risk (OR = 1.66) for having a bipolar disorder than the total sample of non-BPD personality disorders.

## Discussion

### Prevalence of Affective Disorder in General

The frequency of affective disorders was generally lower for BPD than in the aggregate sample of other personality disorders. This is in contrast to the findings of Zanarini et al. [5], who found that affective disorders were more frequent among subjects with BPD compared to a sample having other personality disorders, and to Fyer et al. [21], who found no such difference. Both the Zanarini et al. and Fyer et al. samples consisted of inpatients that are expected to have a more severe psychiatric symptom load than outpatients. This may explain the lower incidence of affective disorders in the present study. Different diagnostic assessment methods may also contribute to differing results.

The prevalence of affective disorders in the BPD group in our study was higher than among persons in the general population [26]. This is not surprising, since the sample in this study were patients receiving specialized psychiatric treatment. Affective disorders are among the most frequent disorders in mental health services [24]. It is reasonable to assume that persons with personality disorder seeking treatment have a higher rate of affective disorders than both persons with personality disorder and the general population not currently receiving psychiatric treatment.

In the total sample (n = 3 679), depression was five times more frequent than bipolar disorder, and almost nine times more frequent than dysthymia. This indicates that the affective disorders category primarily measures the incidence rate of depression. Variations in the incidence rates for specific affective disorders are therefore difficult to identify and the results may be somewhat misleading. This should be considered in future studies.

### Prevalence of Depression

There was a lower rate of depression in the BPD group than among those with avoidant, dependent, unspecified personality disorders as well as among the total group of non-BPD personality disorders. This is in contrast to earlier studies showing no differences in the rate of comorbidity of depression between persons with BPD and avoidant personality disorder [4] and between persons with BPD and a total group of non-BPD personality disorders [22]. Somewhat unexpectedly we found that the occurrence of depression was lower among patients with BPD than the aggregated sample of other personality disorders. This was unexpected because the BPD group were younger, had a higher ratio of females, a lower level of education, and were less likely to be living with a partner compared to patients suffering from other personality disorders. All these variables represent risk factors for affective disorders [26]. This finding can be understood in several ways. One interpretation is that having a BPD may reduce the risk of developing unipolar depression. It is, however, more likely that the low occurrence of depression among the BPD patients may reflect overlapping diagnostic criteria for depression and BPD, i.e. the clinicians fail to attribute depression symptoms to a unipolar depression because the symptoms are already contextualized to or allocated to the BPD. Prior studies using structural diagnostic assessment methods have found substantially higher point prevalence of depression in patients with BPD [5] compared to the results of this study, which supports this interpretation.

The comparisons between BPD and paranoid, schizoid, dissocial, histrionic and obsessive-compulsive personality disorders yielded no significant differences in the rate of depression. These results verify earlier findings regarding the obsessive-compulsive [4] and the dissocial personality disorders [23]. The fact that patients with BPD did not show a higher rate of depression compared to the majority of patients with other personality disorders suggest that the relationship between BPD and depression is not unique or disorder-specific. Other studies support this understanding in several ways. First, phenomenological differences related to depressive symptoms have been found in depressed patients with and without BPD [27]-[29]. Second, recovery from depression does not predict recovery from BPD [20]. Third, patients with both depression and BPD show less improvement with antidepressant treatment than patients who have depression without BPD [29],[30]. Fourth, neurobiological findings suggest that the affective instability in BPD is characterized by a strong reactivity to environmental cues and interpersonal stressors, while affective instability in depression appears to be characterized by autonomous mood changes [31]. However, several studies support the notion that BPD and depression share several etiological factors. For example, Gunderson et al. [20] observed that the presence of BPD extends the duration of depression, and that improvement in BPD predicts improvement in depressive symptoms. Fava et al. [6] found that early onset depressions were more often linked to cluster B in general and to BPD in particular, than late onset depressions. Janzing et al. [32] noted that patients with early onset depressions are more likely to have a chronic course and a positive family history of affective disorder, than patients who develop depression later in life. These findings suggest a stronger pathogenetic component in the early onset group, and that BPD patients and patients with early onset depression share several of these components.

### Prevalence of Bipolar Disorder

Compared to each personality disorder separately, the BPD group did not differ significantly in prevalence of bipolar disorder. BPD had a numerically higher rate, apart from the dissocial and obsessive-compulsive types. However, BPD was significantly more likely to have bipolar disorder compared to the aggregate sample of non-BPD personality disorders. The results indicate a tendency for BPD to be associated with bipolar disorder more often than other personality disorders.

BPD is a personality disorder in which the key characteristics are unpredictable and rapidly shifting mood, impulsivity and lack of self control [25]. These symptoms are also found in bipolar disorder. It may be considered surprising that BPD patients did not show a higher rate of bipolar disorder compared to patients with other personality disorders where such traits are less prominent, such as the schizoid, avoidant and dependent types. Numerically, the prevalence in the latter personality disorders is half the prevalence of bipolar disorder in BPD. Nonetheless, this difference does not reach statistical significance due to small numbers. On this point then, the findings are in accordance with McGlashan et al. [4] who also found no significant differences when comparing patients with BPD to the schizotypal, avoidant and obsessive-compulsive types with regard to frequency of bipolar disorder.

Several researchers are open to classify BPD as a mood disorder rather than a personality disorder [12],[13],[33]. Akiskal [12] points to the shared phenomenology and family history as evidence that BPD should be considered a part of the bipolar spectrum. Furthermore, he points out that patients with BPD often report irritability and hypomania as adverse effects of antidepressants, and they benefit from mood-stabilizing medication. Hirschfeld and Cross [34] found an earlier age of onset for those who develop bipolar disorder than for those who develop unipolar disorder. Fava et al. [6] observed that patients with BPD have a particular tendency toward an early onset of affective disorders compared to patients with other personality disorders. Seen together, Hirschfeld and Cross’ and Fava et al.’s findings might suggest that the co-occurrence between BPD and bipolar disorder may have a stronger biological component than the co-occurrence of BPD and unipolar depression. Thus, it may be assumed that there are shared biological and/or genetic factors for BPD and bipolar disorder.

Although other research suggests that the relationship between BPD and bipolar disorder has a stronger biological component than for BPD and unipolar depression, it does not necessarily follow that BPD is a bipolar spectrum disorder. There may be several reasons for this. First, only 5.7% of the BPD patients in this study had bipolar disorder. Second, neurobiological studies propose that affective instability in BPD is characterized by reactivity to psychosocial cues, while affective instability in bipolar disorder primarily is internally driven [31]. Third, there are phenomenological differences between the two disorders. For example, Benjamin and Wonderlich [27] found that patients with BPD and patients with bipolar disorder have diametrically opposite views of relationships (hostile and autonomous vs. non-hostile and interdependent). Wilson et al. [35] noted that patients with bipolar disorder and BPD have higher rates of several cognitive symptoms than do patients who have bipolar disorder without BPD. Finally, it is found that while affective instability is characteristic of both BPD and bipolar disorder, only BPD is associated with impulsivity [17],[36]. Although impulsivity is typical of mania, Lewis, Scott and Frangou [37] concluded that this was a state and not a trait marker of bipolar disorder, since they found no increased impulsivity among patients with bipolar disorder in remission. It is therefore possible that impulsivity differentiates BPD from bipolar disorder. However the fact remains that the increased risk for bipolar disorder in BPD indicates some kind of association between the two disorders. This association may be due to an etiological relationship, or it may be evidence of overlapping diagnostic criteria, or both.

BPD is traditionally considered as chronic and a hard-to–treat disorder. Recent research has revealed that a significant number of patients with BPD respond to treatment [38] and achieve remission over time [39],[40]. Nevertheless, patients with BPD have more difficulties obtaining and sustaining remission and recovery, and have shorter remissions, than patients with other personality disorders [40]. Akiskal [12] questioned whether clinicians over diagnosed patients with atypical and unstable affective disorders as BPD. A recent review by Coulston et al. [33] reported that rapid cycling bipolar disorder and BPD had more overlapping phenomenological and etiological features than bipolar I and II. This highlights our need for more knowledge about these disorders. Consequently, the current diagnostic criteria for affective disorders and BPD are not well enough differentiated, which might contribute to inaccurate diagnosis in the clinic. The results from this study may be a reflection of this.

### Prevalence of Dysthymia

We did not find a higher rate of dysthymia among patients with BPD compared to the other personality disorder groups. This supports the findings of those few studies that earlier have examined this question [4],[22]. From a phenomenological perspective, it may not be surprising that subjects with schizoid and avoidant PD had higher rates of dysthymia than subjects with BPD.

### Other Findings

The frequency of coexisting substance use disorders among patients with BPD is lower than what has been found in other studies [5]. A possible explanation is the organization of the Norwegian community mental health service where patients with substance abuse and psychiatric comorbidity frequently are treated in separate addiction clinics instead of mental health services. Using routine clinical assessment, clinicians are found to identify fewer diagnoses compared to research projects systematically using structural diagnostic methods [41]. Thus, the relatively low incidence of substance use disorders found in this study may be due to this.

In our sample of subjects with non-BPD personality disorders, as many as 80% had either an avoidant, dependent or unspecified personality disorder. The varied selection therefore primarily became a measure of the incidence of affective disorders among these three personality disorders. Certain types of personality disorders are quite rare. This is a common finding in both clinical [7],[42] and non-clinical [22],[43],[44] samples of personality disorders. It is important to maintain this perspective in future research seeking to examine disorder-specific factors in BPD in comparison with other personality disorders.

### Limitations

First, diagnostic assessment is not consistently based on systematic use of structural assessment methods. Since axis II-disorders are found to be under-diagnosed by routine clinical assessment [41] it is reasonable to expect that there are persons with an as yet undiagnosed personality disorder and therefore are not included in the study. We have no information about this group. Although structured diagnostic tools are used to a varying degree in clinical practice, a study by Westen [45] showed that most clinicians carry out thorough examinations of personality disorders and diagnose a single personality disorder, listing this as the primary cause of the patient’s problems. Personality assessments from research-based diagnostic tools often reveal that one patient may have several different personality disorders and only rarely is a single one of them considered the primary diagnosis. The advantage of clinical assessment, as used in this study, is the increased probability that groups will consist of patients who have the personality disorder in question as their primary disorder. This may in fact improve the internal and external validity of the study.

Second, we do not know how clinicians have assessed affective symptoms in various personality groups. Affective disorders may have been diagnosed to a lesser extent in BPD patients than the ones suffering from other personality disorders because BPD often presents with atypical affective symptoms [12],[13], which may be difficult to ascertain. Mood swings are symptomatic of BPD, and clinicians may consider this to be a component of BPD, thereby failing to diagnose a coexisting affective disorder.

Third, whether other axis I or axis II disorders are acting as confounding variables has not been possible to determine, since a maximum of two diagnoses were recorded per patient.

At last, the study is based on the Norwegian population of adult patients receiving outpatient psychiatric care. The results may not apply to patient populations outside of Norway, to more seriously ill inpatients or persons with personality disorders who are not in a treatment setting.

### Summary and Conclusions

This study found that subjects with BPD had a lower rate of affective disorders than an aggregated sample of subjects with other personality disorders. However, the rate varies somewhat among the groups of specific personality disorders. The findings regarding specific types of affective disorders revealed a disassociation, in that the rates of unipolar depression and dysthymia were lower among subjects with BPD than among subjects with several other personality disorders, whereas the rate of bipolar disorder tended to be higher. This suggests a stronger association between BPD and affective disorders in the bipolar spectrum than those in the unipolar spectrum. This association may reflect an etiological relationship or diagnostic overlapping criteria, or both.

To the authors’ knowledge this is the first study that compares the occurrence of different types of affective disorders in BPD to the entire spectrum of each individual personality disorder simultaneously. Further studies using structured diagnostic instruments are needed to confirm the results of this study. There is an overall need for studies addressing the relationships between the phenomenological, biological, pathogenetic and treatment-related aspects of BPD and other specific personality disorders, as well as patients with affective disorders without personality disorders. Such studies would help us identify which aspects of the interaction between BPD and affective disorders can be described as a general consequence of having a personality disorder, and which aspects of the interaction between BPD and affective disorders are type specific. This is clinically relevant since it may give mental health professionals the opportunity to offer a more precise and evidence based treatment to patients with BPD and coexisting affective disorders.
